# Discovery and Genetic Validation of Chemotherapeutic Targets for Chagas' Disease

**DOI:** 10.3389/fcimb.2018.00439

**Published:** 2019-01-07

**Authors:** Juan Felipe Osorio-Méndez, Ana María Cevallos

**Affiliations:** ^1^Laboratorio de Microbiología y Biología Molecular, Programa de Medicina, Corporación Universitaria Empresarial Alexander von Humboldt, Armenia, Colombia; ^2^Grupo de Estudio en Parasitología Molecular, Centro de Investigaciones Biomédicas, Universidad del Quindío, Armenia, Colombia; ^3^Departamento de Biología Molecular y Biotecnología, Instituto de Investigaciones Biomédicas, Universidad Nacional Autónoma de México, Mexico City, Mexico

**Keywords:** *Trypanosoma cruzi*, chagas disease, chemotherapautics, drug discovery, drug validation

## Abstract

There is an urgent need to develop new treatments for Chagas' disease. To identify drug targets, it is important to understand the basic biology of *Trypanosoma cruzi*, in particular with respect to the biological pathways or proteins that are essential for its survival within the host. This review provides a streamlined approach for identifying drug targets using freely available chemogenetic databases and outlines the relevant characteristics of an ideal chemotherapeutic target. Among those are their essentiality, druggability, availability of structural information, and selectivity. At the moment only 16 genes have been found as essential by gene disruption in *T. cruzi*. At the TDR Targets database, a chemogenomics resource for neglected diseases, information about published structures for these genes was only found for three of these genes, and annotation of validated inhibitors was found in two. These inhibitors have activity against the parasitic stages present in the host. We then analyzed three of the pathways that are considered promising in the search for new targets: (1) Ergosterol biosynthesis, (2) Resistance to oxidative stress, (3) Synthesis of surface glycoconjugates. We have annotated all the genes that participate in them, identified those that are considered as druggable, and incorporated evidence from either *Trypanosoma brucei, and Leishmania* spp. that supports the hypothesis that these pathways are essential for *T. cruzi* survival.

## Introduction

Chagas' disease, leishmaniasis, and human African trypanosomiasis are the three main parasitic diseases caused by flagellated protists of the order Kinetoplastida. Chagas' disease is caused by *Trypanosoma cruzi*, and is considered as one of the most prevalent parasitic diseases worldwide. Chagas' disease is present mainly in rural and peri-urban areas of Latin America, although migration has expanded its distribution to non-endemic countries. *T. cruzi* is a parasite that infect multiple species of triatomine hematophagous bugs and several mammalian hosts, including humans. At least four stages have been recognized during the *T. cruzi* life cycle: epimastigotes, amastigotes and metacyclic, and bloodstream trypomastigotes (Tyler and Engman, 2001). Epimastigotes replicate extracellularly in the gut of the insect vector, where they differentiate into non-replicative and highly infective metacyclic trypomastigotes. Parasites at this stage are delivered by the feces of triatomines during a blood meal from a mammalian host, and reach the mucosa or the bloodstream through a vulnerable region of the skin. There, the parasite invades nucleated cells and differentiates into the amastigote stage, which replicates inside the cytoplasm of the host cell. Then, the parasite egress from the host cells as bloodstream trypomastigotes that may invade additional cells to proliferate to other tissues or be transmitted to a new triatomine vector. Other modes of transmission to the human include congenital and oral infection, and blood transfusion or organ transplantation from infected donors. In humans, the infection starts with an acute phase that lasts 4–8 weeks. The host's immune response typically control the parasite replication, but is not capable of clearing the infection. This leads to the chronic phase of the disease, in which the parasite persists intracellularly mainly in the heart, skeletal muscles, and gastrointestinal tissues. Around 30% of the chronically infected people develop serious cardiac alterations, and up to 10% suffer neurological, digestive, or mixed disorders (Nagajyothi et al., 2012). The mechanisms involved in parasite persistence are not known. However, recently it has been suggested that a form of “dormant” amastigotes may be involved (Sánchez-Valdéz et al., 2018).

The efficacy of the two chemotherapeutic agents of current use (nifurtimox and benznidazole) for the treatment of Chagas' disease is highly variable and often limited, especially during the chronic phase of the infection (Urbina, 2010). Both drugs require long periods of administration and have significant side effects that frequently force the physician to stop treatment (Castro et al., 2006). Furthermore, resistant strains have also been reported (Filardi and Brener, 1987; Bern, 2011). Most significantly, the recently identified dormant forms of the parasite were resistant to extended drug treatment *in vivo* and *in vitro* and remain able to re-establish infection after as many as 30 days of drug exposure (Sánchez-Valdéz et al., 2018). Thus, there is an urgent need to develop new treatments that are safe and of low cost. In this work, we discuss the characteristics required for a drug target to be useful and review the candidate genes and pathways that have been genetically or pharmacologically validated as essential and druggable in *T. cruzi* and incorporate the data that is available from *T. brucei* and *Leishmania* spp.

## Identification of New Treatments for Chagas' Disease

The first stage for the discovery or repurposing of antimicrobial agents is target identification. It usually involves the screening of collections of compounds against a molecular target, typically an enzyme (target-based screening), or against whole organisms (cell-based or phenotypic screening). All candidates must then be refined through a cyclic process of structure modifications, until they achieve significant activity, typically in an animal model of infection. Subsequently, the biological activity, pharmacokinetics, and safety profile of the series are optimized by a process that leads to the selection of candidate drugs. Selected drugs are then submitted to a process of regulatory toxicology and scale-up that enables their evaluation in human studies (De Rycker et al., 2018). Unfortunately, the probability of a drug entering the clinical testing phase and its eventual approval is only about 12%, with an estimated out-of-pocket cost per approved new compound of US $ 1,395 million (DiMasi et al., 2016). Because of the cost of development of new drugs, the relatively limited target population and the economic power of the countries where Chagas' disease is endemic, the majority of pharmaceutical companies have shown little interest in the development of new drugs for the treatment of parasitic diseases (Tarleton, 2016). In the absence of adequate funding it is vital to design research projects that take advantage of available biological, bioinformatic, structural, and chemical data that is being incorporated in large publicly available databases.

It has been recognized that to obtain a successful new treatment it is important to understand, from the outset, the essential attributes (target product profile) required for a specific drug to be a clinically successful product and substantially better than the existing therapies (Wyatt et al., 2011). The ideal target product profile for Chagas' disease was defined as a drug that is effective in both acute and chronic disease, that it is active against all strains and at all ages, with a clinical efficacy superior to benznidazole (Chatelain, 2015). It should be administered orally (once a day for 30 days) and require no clinical evaluation, laboratory testing nor need for electrocardiograms during treatment. It should not have any contraindications or interactions with other drugs and lack genotoxicity, teratogenicity, inotropic effect, and proarrhythmic potential. There are many challenges to achieve such goals, including the selection of the chemical compounds and the suitability of the chosen target. To help in this goal, criteria for the selection of suitable targets have been suggested (Wyatt et al., 2011). Among them are essentiality, druggability, available structural information, and selectivity over the host's orthologs. Here, we discuss bioinformatic and experimental strategies to identify and validate chemotherapeutic targets based on them.

### Essentiality

Essentiality refers to genetic and chemical evidence that the target is indispensable for growth or survival. Ideally, proteins that are essential for survival in the parasite stages present in the host should be selected. To validate them is necessary to demonstrate that the disruption or deletion of such genes causes cell death. In trypanosomatids, studies on the essential role of genes for cell viability are usually conducted in *T. brucei*. This species have a RNA interference (RNAi) system that is widely used to generate gene knock-downs in an inducible manner. The induction of the RNAi against an essential gene product leads to cell death, and thus to a rapid inhibition of cell proliferation. In *T. brucei* this strategy is so efficient that has even been used for a genome-scale screening for essential genes (Alsford et al., 2011). *T. cruzi* lacks a functional RNAi pathway (Kolev et al., 2011), so it cannot be used. An alternative would be extrapolate the huge amount of information obtained in *T. brucei* to *T. cruzi*. However, this is not possible when a given gene of *T. cruzi* has not an orthologous counterpart in *T. brucei*. Also, essentiality may depend on the species or on the developmental stage of the parasite. As an example, in *T. brucei*, a highly conserved protein such as actin is essential in the vertebrate bloodstream stage but not in the insect procyclic stage (García-Salcedo et al., 2004). Thus, essentiality must be evaluated for each species and at the relevant developmental stages of the parasite. In *T. cruzi*, this has only been accomplished by gene knock-out in epimastigotes, the insect stage of the parasite (see Burle-Caldas et al., 2015 for a review). In this strategy, the endogenous copy of the gene is replaced by a selectable marker using homologous recombination. To evaluate other parasite stages it is necessary to differentiate transgenic epimastigotes or infect cells or animal models with the transformed parasites. There are several limitations with this approach. When there is more than one copy of the gene, several replacements are required to obtain null-mutants. Thus, the essentially of multicopy genes or those within multigenic families is difficult to evaluate. On the other hand, deletion of essential genes often leads to unviable cells, so null-mutants cannot be selected to analyze the resulting phenotypes. *T. cruzi* is a diploid organism, and in some circumstances only a single copy of an essential gene may be needed (haplosufficiency), with the heterozygote displaying an instructive phenotype. However, when this is intolerable, an inducible, or transient copy of the gene has to be introduced before the gene replacement (Jones et al., 2018). As a reflection of these difficulties, only 16 *T. cruzi* genes have been shown to be essential in reverse genetic experiments (Table 1). An alternative to the mentioned traditional methods to evaluate essentially is the use of inducible approaches such as Di-Cre or the DHFR degradation domain, or genome edition tools such as CRISPR-Cas9 or Zinc-finger endonucleases. These systems have been adapted to *T. cruzi* in the last 5 years (Kangussu-Marcolino et al., 2014; Peng et al., 2014; Lander et al., 2015; Ma et al., 2015; Burle-Caldas et al., 2017), although recent work has focused on the optimization of different CRISPR-Cas9 configurations (e.g., Beneke et al., 2017; Lander et al., 2017; Soares Medeiros et al., 2017; Burle-Caldas et al., 2018; Costa et al., 2018). The future widespread use of CRISPR-Cas9 will help overcome the current technical limitations for the genetic manipulation of *T. cruzi*. It will allow the rapid evaluation of phenotypes resulting from the disruption of essential genes in different stages of the parasite, including those expressed as multicopy genes and within multigenic families.

**Table 1:** List of published essential *T. cruzi* genes as identified by Jones et al. (2018).

**Criteria of essentiality^a^**	**Gene**		**Protein**	**Biological process**	**Druggability score**		**Structure TDR**	**Inhibitors TDR**
					**TP^d^**	**TDR^d^**	
Failed 2KO recombination	TcCLB.503527.40	*GPI10*	GPI alpha-mannosyltransferase III	GPI-anchor biosynthesis	N.D.	N.D.	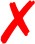	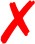
	TcCLB.511481.40	*GPI12*	N-Acetyl-D-glucosaminylphospha-tidylinositol de-N-acetylase	GPI-anchor biosynthesis	0.302	N.D.	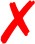	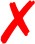
	TcCLB.503419.30	*TC52*	Thioredoxin-glutaredoxin like	Glutathione-mediated detoxification	0.881	N.D.	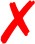	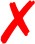
	TcCLB.507091.40	*DHOD*	Dihydroorotate dehydrogenase	‘De novo' pyrimidine nucleobase biosynthesis	0.758	N.D.	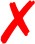	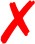
	TcCLB.506811.190	*GALE*	UDP-glucose 4′-epimerase	Galactose metabolism, UDP-galactofuranose biosynthesis	0.238	0.1	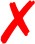	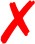
	TcCLB.509153.90	*DHFR-TS*	Dihyrofolate reductase—thymidylate synthase	Folate metabolism	0.715	0.8	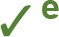	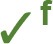
	TcCLB.507547.40	*ECH1*	Enoyl-coenzyme A (CoA) hydratase 1	Fatty acid beta oxidation	0.459	N.D.	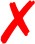	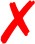
	TcCLB.508319.40	*SUB2*^b^	RNA helicase DEAD-box protein	RNA polymerase I core binding; mRNA metabolism	0.270	N.D.	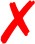	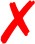
	TcCLB.511277.450	*GPI8*	Cysteine peptidase, Clan CD, family C13, putative	GPI-anchor biosynthesis	Not found	N.D.	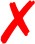	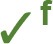
	TcCLB.509011.40	*CRT*	Calreticulin	Binding to misfolded protein	0.000	N.D.	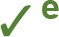	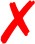
	TcCLB.509461.90	*IP3R*^**b**^	Inositol 1,4,5-trisphosphate receptors	Transmembrane transport	0.875	0.7	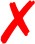	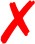
	TcCLB.510821.50	*RPA2*	Replication protein A	DNA replication and repair	Not found	N.D.	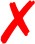	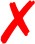
	TcCLB.509757.30	*STI1*	Stress-inducible protein 1	Protein binding	0.719	N.D.	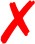	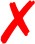
2KO recombination + episomal copy	TcCLB.511283.90	*NMT*	N-myristoyltransferase	N-terminal protein myristoylation	0.753	0.6	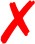	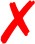
Failed 2KO CRISPR-CAS9	TcCLB.511353.30	*?*^c^	Triose-phosphate transporter	Transporter	0.727	N.D.	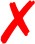	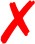
1KO CRISPR-CAS9 + Posaconazole S	TcCLB.510101.50	*CYP51*	Lanosterol 14-alpha-demethylase	Ergosterol biosynthesis	0.968	0.8	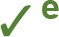	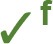

a *Criteria of essentiality: Failed 2KO recombination, double knock out were not obtained by targeted disruption by homologous recombination. 2KO recombination + copy, double knock out was obtained but only in the presence of an episomal copy of the gene; Failed 2KO, the double knock out was not obtained using CRISPR-Cas9, 1 KO CRISPR-Cas9 a single knockout was obtained with increased sensitivity to the specific inhibitor posaconazole*.

b *The Gene ID identified in the Jones et al. (2018) study correspond to an allele for which the complete sequence is not available. The Gene ID for the corresponding haplotype (with complete sequence) is used instead*.

c *TcCLB.510821.50 is essential but its characterization as GALF is not. TcGalf is annotated as UDP-galactopyranose mutase (Oppenheimer et al., 2011), the targeted gene is a putative transporter that has not been characterized*.

d *Druggability score: TP-found in the Target-Pathogen Database, TDR-found in the Tropical Disease Research Database. Green numbers indicate proteins classified as highly druggable and red numbers those classified within other categories*.

e *Structures identified at the TDR database*.

f *Inhibitors identified at the TDR database*.

*N.D. indicates genes are included in the database but are not scored. Not found indicates that the gene has not been included in the database*.

#### Selectivity

Selectivity depends on the target not being either present in the host, or being highly modified or not essential for its survival. High affinity and selectivity are two of the characteristics of a drug for its target that are routinely sought in the search of new therapies. Selectivity can be difficult to achieve, especially for targets that belong to large families of structurally and functionally related proteins. Therefore, targets that are present in the parasite and not in the host would be preferred. If a homolog is present in humans, targets that differ the most would be favored. In general, selectivity can be improved during drug optimization by modifying the compound improving its affinity toward the parasite target to a higher extent than it does to the human homolog (Kawasaki and Freire, 2011). To achieve this, it is necessary to have structural information of both parasitic and human proteins. At October 2018 there are 286 *T. cruzi* structures deposited at the Protein Data Bank (https://www.rcsb.org/). Nearly half of them correspond to only six proteins that are considered as promising drug targets: 58 of Dihydroorotate dehydrogenase, 26 of Cruzain, 21 of Lanosterol 14-alpha-demethylase, 10 of Dihydrofolate reductase-thymidylate, 15 of Farnesyl diphosphate synthase, and 6 of Squalene synthase. Thus, structural information of other *T. cruzi* proteins is urgently needed.

#### Druggability

Druggability describes the ability of a protein to bind a drug-like molecule, which in turn modulates its function in a desired way. Druggable proteins should have a well-defined pocket with suitable physicochemical attributes to allow drug binding-sites prediction (Sosa et al., 2018). The druggability of a target is usually estimated by comparing it with homologs in other organisms that have been successfully targeted with specific drugs. Another way to assess druggability is by the development of mathematical algorithms that use structural information about a protein's binding site to estimate its potential as a target. Several databases that contain such information are freely available. Two of the databases that estimate the druggability for *T. cruzi* proteins are: TDR Targets (Magariños et al., 2012, http://tdrtargets.org/) and Target-Pathogen database (Sosa et al., 2018, http://target.sbg.qb.fcen.uba.ar/patho/). TDR stands for Tropical Disease Research, and it is part of a special program within the World Health Organization that include several tropical pathogens. The database gathers information from multiple sources and published studies including essentiality, functional, and structural information, pathway classification, and information of compounds used to target them. Target-Pathogen database was designed and developed to integrate and give specific weight to protein information (e.g., function, metabolic role, druggability, and essentiality) to facilitate not only the identification of candidate drug targets in pathogens, but also its prioritization. The algorithms employed by both databases are different and therefore discrepancies in the results are likely. The druggability index in both databases is measured in a scale that goes from 0 to 1 and divided into four categories: non druggable (≤0.2), poorly druggable (0.2–0.5), druggable (0.5–0.7), and highly druggable (>0.7) (Sosa et al., 2018).

## TriTrypDB as a Tool for Target Selection

TriTrypDB (http://tritrypdb.org) is an integrated genomic and functional genomic database for pathogens of the family Trypanosomatidae, including organisms in both *Leishmania* and *Trypanosoma* genera. TriTrypDB is the result of continuous collaborative efforts between EuPathDB, GeneDB, and the Seattle Biomedical Research Institute (Aurrecoechea et al., 2017; Warrenfeltz et al., 2018). EuPathDB release 40 (https://eupathdb.org/eupathdb/) contains information about 330 genomes of 321 species that include eukaryotic parasites, relevant free-living non-parasitic organisms and selected pathogen hosts. The current version of the *T. cruzi* CL Brener genome, that was the first sequenced parasite strain (El-Sayed et al., 2005), identifies 21,702 genes. Of them, 13,325 (61%) have a deduced function from its similarity to known genes, and 8,377 (39%) are hypothetical and are therefore of unknown function. Basic research is needed to understand the function and structure of these genes as they may be essential and druggable. Unfortunately, because at least 50% of the *T. cruzi* genome corresponds to repetitive sequences, there are DNA sequence fragments that have not been assembled. Because this lack of sequence disrupts bioinformatics algorithms used to identify open reading frames, many genes are annotated as two or more independent open reading frames (863 genes are annotated as fragments). EuPathDB makes it easy to search for biological questions relating to issues such as stage-specific expression, and to compile lists of genes that share multiple characteristics. As the database includes information about the presence of orthologs and paralogues, it can be easily compared not only across data sets but also across organisms.

## Candidate Genes That Have Been Genetically Validated as Essential in *T. cruzi*

Ideal drug targets should be essential at least in the parasite stages that are present in the host. However, the number of *T. cruzi* genes that have been successfully deleted or disrupted is very small. In a recent review Jones et al. (2018) were able to identify reports where the double knockouts of 20 genes were obtained, demonstrating that these genes were not essential. They also identified papers where the double knockout were not possible to obtain suggesting that the 16 genes studied are essential for epimastigotes (Table 1). We reviewed the available information about these genes to evaluate their potential as drug targets. Based on transcriptomics data available from the TriTrypDB, we found evidence that support the expression of the 16 genes in all stages of the parasite (Li et al., 2016; Supplementary Figure 1). Druggability scores were identified in 13 of the 16 genes, with eight of them scored as highly druggable (score >0.7) and five as poorly or non-druggable (score <0.5) (Table 1). Only three of the encoded proteins have available structures. We then selected three biological pathways known to be important for parasite survival: (1) Ergosterol biosynthesis, (2) Resistance to oxidative stress, and (3) Synthesis of surface glyconjugates. We describe the genetic evidence for the essential role for each gene in the pathway in *T. cruzi* or in other trypanosomatid species. We also compare the pathway with its human counterpart in order to identify enzymes whose inhibition might be selective for the parasite. Finally, we describe the chemotherapeutic agents that target each pathway. This information would help identify other points of these pathways that may be chosen for further study. Other aspects of discovery of new treatments for Chagas disease have been recently reviewed in Field et al. (2017); Francisco et al. (2017); Chatelain and Ioset (2018) and Scarim et al. (2018).

### Ergosterol Biosynthesis

**Figure 1:**
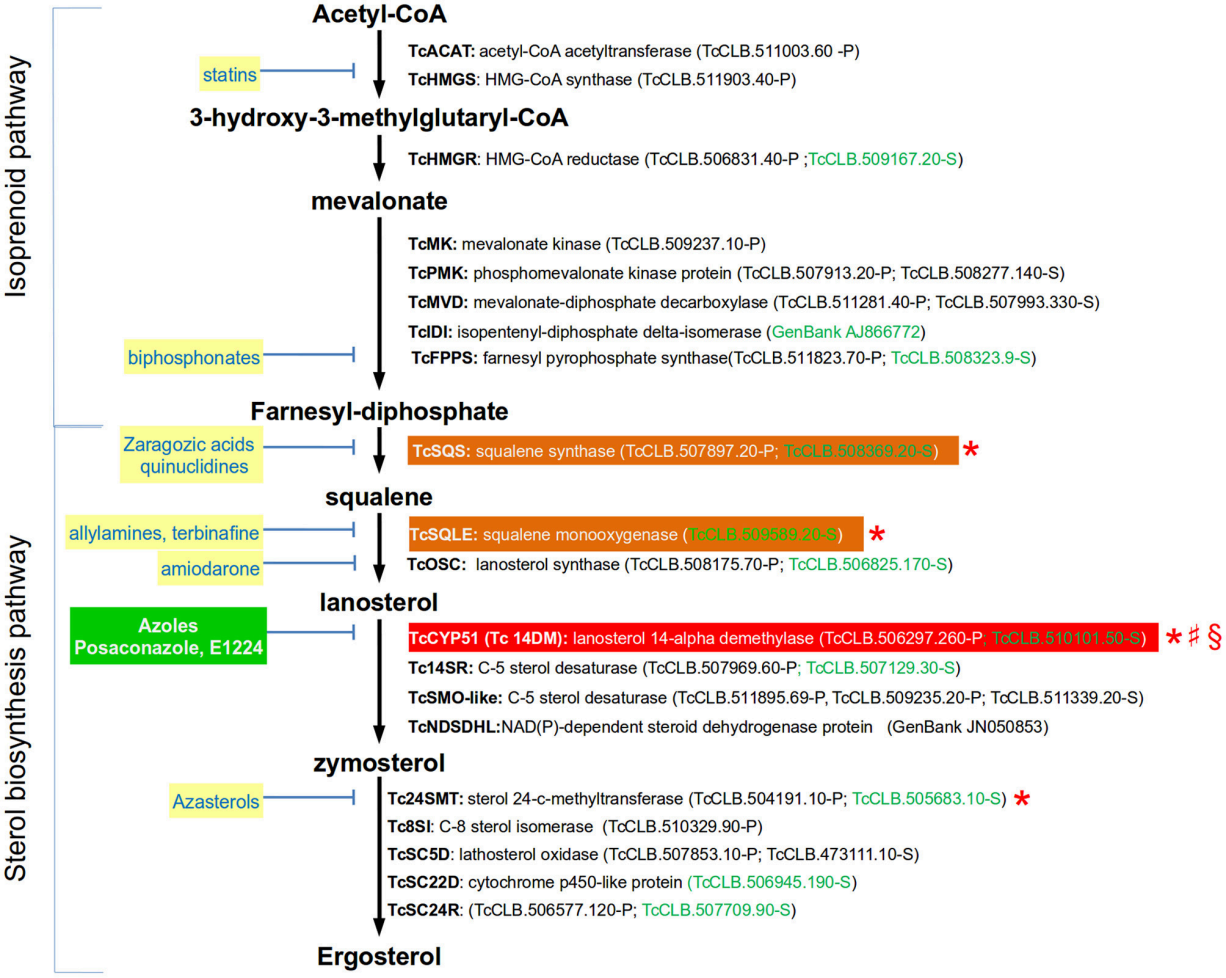
Simplified ergosterol biosynthesis pathway in *T. cruzi* epimastigotes. Gene names are annotated with bold letters, followed by their current annotation in the TriTrypDB. In brackets are the *T. cruzi* locus identifiers of complete genes, followed by the haplotype to which they belong (P = Non-Esmeraldo; S = Esmeraldo). Sequences that have been identified as incomplete or pseudogenes are not included. In the case of TcIDI and TcNDSDHL complete sequences can be found in GeneBank and their accession numbers were used in these cases. Sequences classified as highly druggable (druggability index >0.7) according to the Target-Pathogen database (http://target.sbg.qb.fcen.uba.ar/patho/) are shown with green letters. Known drug classes that block enzymes within this pathway are identified by yellow boxes with blue letters. The only inhibitors that have gone clinical evaluation are shown in a green box with white letters. In a red box is highlighted the enzyme known to be essential in *T. cruzi*, and in orange boxes are highlighted enzymes that are essential in other trypanosomatids. Enzymes that have been genetically manipulated to evaluate their potential role as therapeutic targets in *T. cruzi* (§), *T. brucei* (*), or *Leishmania* (♯) have been annotated with a symbol.

Sterols are lipids produced by all eukaryotic cells that are essential for several processes, including the organization and function of cell membranes. The sterols being synthesized by an organism may vary according to the taxonomic group (Bloch, 1983). In trypanosomes, the main sterol component of the parasite is ergosterol (reviewed in de Souza and Rodrigues, 2009). In *T. cruzi*, endogenously produced ergosterol is a vital resource for the parasite as it cannot be replaced by sterols scavenged from the host. Additionally, one of the clinically relevant forms of the parasite (i.e., amastigotes) is particularly sensitive to the pharmacological inhibition of this lipid (Urbina et al., 1996; Liendo et al., 1999). Ergosterol is synthesized through a biosynthetic pathway that is divided into two stages (Figure 1): the isoprenoid pathway (from Acetyl-CoA to farnesyl diphosphate) and the sterol pathway (from farnesyl-diphosphate to sterols). Farnesyl diphosphate, which is the last product of the first stage, is also the substrate for enzymes catalyzing the production of ubiquinones (such as Coenzyme Q10), dolichols, heme A, and prenylated proteins. The first committed step for sterol biosynthesis begins with the head-to-head condensation of two molecules of farnesyl diphosphate to produce squalene, a two-step reaction catalyzed by squalene synthase. The complete set of genes encoding for the enzymes involved in both stages have been identified in the *T. cruzi* genome (Figure 1). There is only one gene of this pathway whose requirement for the parasite viability has been evaluated in this species (Table 1). In contrast, it has been tested for four of the orthologous genes encoded by *T. brucei*. As expected, silencing of the two first enzymes of the sterol pathway (*Tb*SQS and *Tb*SQLE) resulted in the depletion of cellular sterol intermediates and end products in procyclic cells (Pérez-Moreno et al., 2012). This was associated with impaired cell growth, aberrant cell morphologies, DNA fragmentation and a profound modification of mitochondrial structure and function. Similarly, silencing of the next enzyme on the pathway (*Tb*CYP51) completely stopped growth in procyclic and trypomastigote forms of the parasite (Haubrich et al., 2015; Dauchy et al., 2016). Furthermore, parasites with decreased expression of the enzyme were less virulent for mice (Dauchy et al., 2016). There is also evidence for the importance of CYP51 in the viability of *T. cruzi* and *Leishmania*. In *T. cruzi*, a combination of CRISPR-Cas9 gene edition and pharmacological inhibition of the enzyme produced a concentration-dependent growth decrease in epimastigotes (Soares Medeiros et al., 2017), suggesting that the function CYP51 is essential for the parasite. In *L. donovani*, genes encoding for this enzyme could only be knocked out in the presence of episomal complementation (McCall et al., 2015). *L. major* CYP51-null mutants were viable but had defects in their growth and had hypersensitivity to heat stress (Xu et al., 2014). Finally, inhibition of the last enzyme of the pathway in *T. brucei* (*Tb*SMT) showed contradictory evidence regarding its role on parasite growth, as in one study it did decreased it and in the other not (García-Salcedo et al., 2004; Haubrich et al., 2015). The reason for this difference has not been clarified, but it is possibly related with the presence of sterols in the used experimental media that might be used for the parasite to spark cell proliferation.

In contrast with trypanosomes and fungi, human cells produce cholesterol instead ergosterol. For this reason, enzymes from the ergosterol biosynthetic pathway are common targets of inhibitors used to treat fungal infections (Figure 1), and some of them have also been tested in trypanosomes. Within the isoprenoid pathway, the HMG-CoA synthase and the farnesyl pyrophosphate synthase are inhibited by statins and biphosphonates, respectively. However, the intervention on this pathway have the disadvantage of affecting the synthesis of downstream isoprenoid compounds that are essential for other metabolic pathways in human cells. Squalene synthase (SQS), squalene monooxigenase (SQLE), lanosterol 14-alpha demethylase (CYP51) and sterol 24-C-methyltransferase (24MST) are the major enzymes targeted for pharmaceutical inhibition in the sterol pathway (Figure 1). Lanosterol synthase (OSC) has been less studied, but its inhibition by amiodarone has shown to affect ergosterol synthesis in *T. cruzi* (Benaim et al., 2006). CYP51 is by far the enzyme of the ergosterol pathway that has been studied in more detail as a therapeutic target for *T. cruzi*. This enzyme uses lanosterol or related compounds as substrates to produce zymosterol, which is the precursor of ergosterol. Azoles are highly effective antifungal drugs that work by inhibiting the activity of CYP51. Several azoles have shown strong anti-*T. cruzi* activity *in vitro* and *in vivo* (Lepesheva et al., 2011), so they have been considered a priority for drug development. The mechanism of action of these compounds against the *T. cruzi* CYP51 has been studied at the structural level (Chen et al., 2010; Lepesheva et al., 2010; Hoekstra et al., 2016). Also, the specificity of inhibition of *T. cruzi*'s CYP51 by posaconazole, a strong anti-fungal azole, has been evaluated using a CRISPR-Cas9 mediated gene edition, which resulted in a 10-fold increase in sensitivity for the drug compared with the wild-type strain (Soares Medeiros et al., 2017). Unfortunately, clinical trials showed that two promising azoles are ineffective to treat chronic Chagas disease in humans. The CHAGASAZOL trial used posaconazole and ravuconazole monotherapy and failed to obtain a sustained parasite clearance after treatment (Chatelain, 2015). Similarly, the STOP-CHAGAS study showed that benznidazole was superior to posaconazole, either as monotherapy or combined, in obtaining a sustained serological response at 6 months in individuals with asymptomatic *T. cruzi* infection (Morillo et al., 2017). Based on these results, it has been suggested that CYP51 inhibition should no longer be considered as a priority for drug development against *T. cruzi* (Sykes and Avery, 2018). However, azoles such as VNI, VNV, and VT-1161 may not discarded as feasible candidates as they showed to be safe, and highly efficient, and selective to eradicate *T. cruzi* infections from murine models (Villalta et al., 2013; Lepesheva et al., 2015; Hoekstra et al., 2016; Guedes-da-Silva et al., 2017). A relatively unexplored and promising strategy is the inhibition of the last enzyme of the ergosterol synthesis (*Tc*24SMT) by azasterols (Figure 1). In contrast to other enzymes of the pathway, *Tc*24SMT is absent in humans. *T. cruzi* parasites treated with the 24SMT inhibitors 22,26-azasterol (AZA) and 24(R,S) 25-epiminolanosterol (EIL) showed not detectable levels of 24-alkyl sterols and strongly inhibited parasite growth (Urbina et al., 1996). Interestingly, amastigotes were more susceptible than epimastigotes to these compounds and synergistic antiparasitic effects of AZA and CYP51 inhibitors were observed *in vitro* and in a murine model of acute Chagas disease. Other 24SMT inhibitors have shown effects on epimastigote growth accompanied by severe ultrastructural alterations (Braga et al., 2005).

In conclusion, enzymes from the ergosterol pathway are common targets of effective inhibitory compounds to treat fungal infections. Ergosterol is endogenously produced and essential for *T. cruzi*, so these molecules have also been considered promising anti-Chagasic drugs. Most studies in this direction have been focused on the inhibition of CYP51 by azoles. Evidence from these works suggested that these compounds were efficient to combat the infection using *in vitro* and murine models. However, they failed when tested in clinical trials. Despite these results, other azoles and inhibitors of distinct enzymes of the pathway, specially *Tc*24SMT, remain as plausible candidates as anti-*T. cruzi* drugs that deserve further exploration.

### Resistance to Oxidative Stress

**Figure 2:**
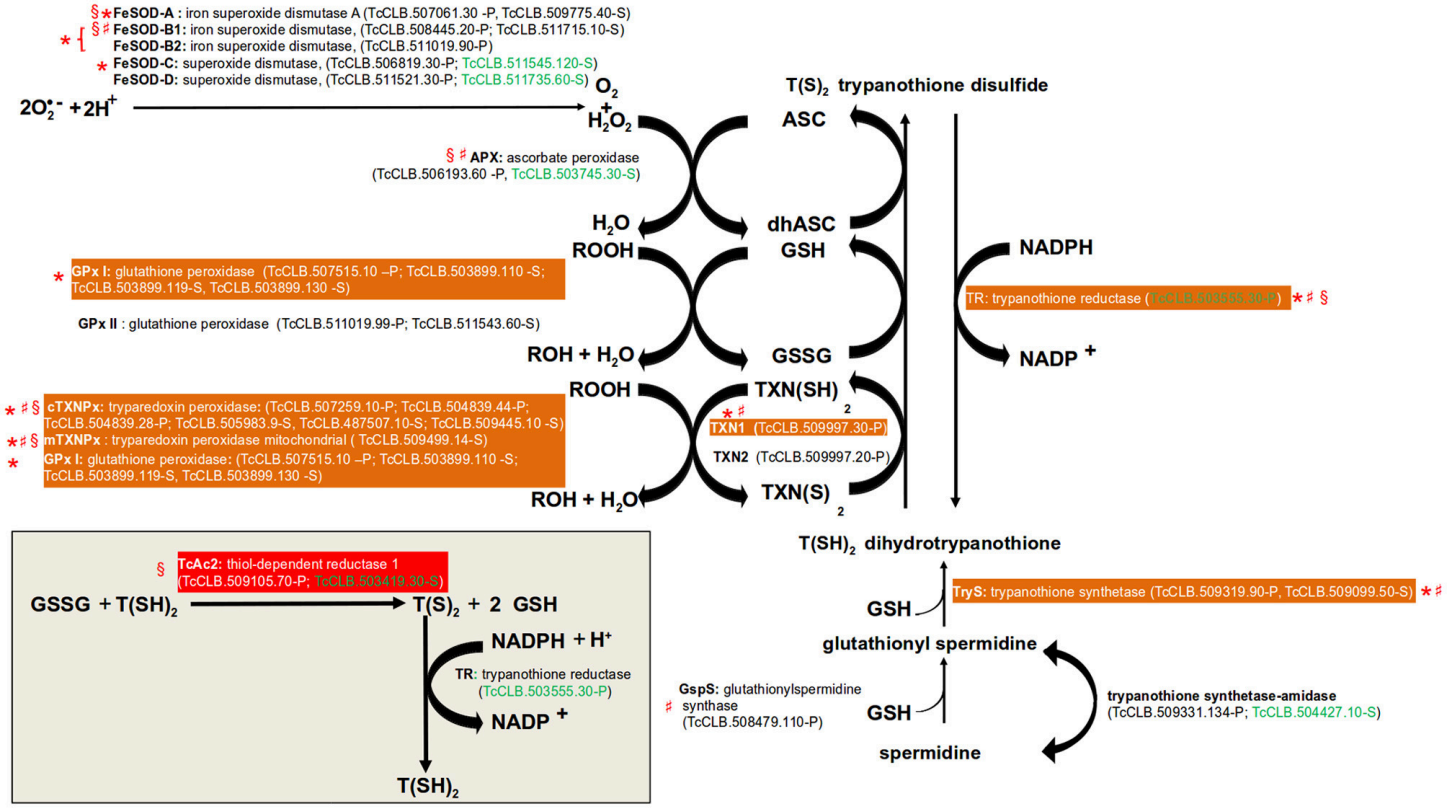
Pathways of defense to oxidative stress. Gene names are annotated with bold letters, followed by their current annotation in the TriTrypDB. In brackets are the *T. cruzi* locus identifiers of complete genes, followed by the haplotype to which they belong (P = Non-Esmeraldo; S = Esmeraldo). Sequences that have been identified as incomplete or pseudogenes are not included. Sequences classified as highly druggable (druggability index >0.7) according to the Target-Pathogen database (http://target.sbg.qb.fcen.uba.ar/patho/) are shown with green letters. In a red box is highlighted the enzyme known to be essential in *T. cruzi*, and in orange boxes are highlighted enzymes that are essential in other trypanosomes. Enzymes that have been genetically manipulated to evaluate their potential role as therapeutic targets in *T. cruzi* (§), *T. brucei* (*), or *Leishmania* (♯) have been annotated with a symbol.

In all organisms, molecular species with high redox potential have important physiological roles (Sies et al., 2017), especially in signal transduction. However, an excessive exposure to them may lead to oxidative stress, which is detrimental to the cells. Therefore, there are antioxidant mechanisms to deal with these situations. Two major forms of them have been described: (1) Enzymatic protection, (2) Low-molecular weight compounds containing thiol groups (R-SH). The first is comprised by enzymes that directly catalyze the reduction of these molecules. In the second category, the thiol group acts as the reducing agent. Within them, the most ubiquitous is glutathione (GSH), a tripeptide composed by Glu, Cys, and Gly. Others include α-tocopherol (vitamin E) and ascorbate (vitamin C) (Poole, 2015). *T. cruzi* is exposed to toxic oxygen and nitrogen species derived from its aerobic metabolism and from the host immune response. To overcome this, the parasite possess an antioxidant system based on enzymatic protection, GSH, and the GSH-containing molecule trypanothione (Turrens, 2004; reviewed in Krauth-Siegel and Comini, 2008; Figure 2). The major enzymatic mechanism expressed by *T. cruzi* are five iron superoxide dismutases (FeSOD, two mitochondrial and three cytosolic) that remove superoxide anions (O_2_^**−**^) by converting them into hydrogen peroxide (H_2_O_2_) and molecular oxygen (O_2_). The H_2_O_2_ produced by the FeSODs, and from other sources, can then be reduced in the endoplasmatic reticulum (ER) to water by the ascorbate peroxidase (Apx) (Wilkinson et al., 2002). This leads to the conversion of ascorbate (ASC) into dehydroascorbate (dhASC). On the other hand, highly reactive hydroperoxides (ROOH) can be reduced by two sets of enzymes: glutathione peroxidases (cytosolic GPx I and the ER located GPx II) and tryparedoxin peroxidases (cytosolic cTXNPx and mitochondrial mTXNPx). Glutathione peroxidases oxidize two molecules of GSH to glutathione disulfide (GSSG). Instead of using GSH, tryparedoxin peroxidases uses the thiol groups of its cysteine residues. Trypanothione [T(SH)_2_] is composed by two GSH molecules bound by a polyamine named spermidine. T(SH)_2_ has a key role in the described trypanosomatid antioxidant mechanism because it non-enzymatically reduces the generated dhASC, GSSG, and TXN(S)_2_. This replenish the parasite with molecules with the potential to keep the system working. The process is assisted by two enzymes that catalyze the reduction of GSSG (Thiol-dependent reductase, TcAc2) (Figure 2, inset) and TXN(S)_2_ (tryparedoxin, TXN1 and 2). The oxidized form of trypanothione [T(S)_2_] is returned back to T(SH)_2_ by the enzyme trypanothione reductase, a reaction that requires NADPH.

The essential role of several of these enzymes has been evaluated in *T. cruzi* and other trypanosomatids. Regarding the FeSODs, there are studies for four of the five isoforms encoded by the parasite genome (FeSOD-A, FeSOD-B1 and FeSOD-B2, FeSOD-C) in *T. brucei* and *Leishmania* (Beltrame-Botelho et al., 2016; Figure 2). In *L. chagasi*, a single-allele knockout of FeSOD-B1 resulted in a decrease of growth when exposed to the O_2_^**−**^ generating agent paraquat (Plewes et al., 2003), suggesting an important role for this enzyme. The mutant parasites also showed a reduced level of survival within macrophages. RNAi studies in the bloodstream forms (BSFs) of *T. brucei* showed that the RNAi mediated knockdown of FeSOD-B1/2, but not for FeSOD-A and FeSOD-C, resulted in a significant reduction in parasite growth (Wilkinson et al., 2006). However, in the presence of paraquat, RNAi inhibition of FeSOD-A resulted in decreased growth (Wilkinson et al., 2006). APx is an antioxidant enzyme that is absent from the mammalian host, but unfortunately it has been shown to be dispensable for the parasite. *T. cruzi* null mutants for the enzyme had no defects on cell growth and were able to complete their life cycle *in vivo* (Taylor et al., 2015). In *L. major*, APx null mutants even shown hypervirulence after infection in macrophages and inoculation into mice (Pal et al., 2010). With the exception of GPx II, all tested glutathione and tryparedoxin peroxidases have shown to be essential for *T. brucei* or *Leishmania* (Figure 2). In *T. brucei*, a knockdown of the GPx I genes was lethal to BSFs (Wilkinson et al., 2003). A similar result was observed for the cytosolic cTXNPx, but not for the mitochondrial mTXNPx (Wilkinson et al., 2003). However, gene replacement studies showed that this last enzyme is essential in *L. infantum* (Castro et al., 2011). As mentioned above, the reduction of GSSG to GSH is thought to be largely dependent on the oxidation of T(SH)_2_. However, in *T. cruzi* TcAc2 is able to catalyze this reaction (Moutiez et al., 1995). Interestingly, this activity seems to be essential for epimastigotes as it was not possible to obtain null mutants for the encoding gene (Allaoui et al., 1999). Tryparedoxins (TXNs) are oxidoreductases found exclusively in trypanosomatids. There are two TXNs in *T. cruzi*, one cytosolic (TXN1) and the other associated with endomembranes (TXN2) (Arias et al., 2013). The essential role of neither of them have been tested in *T. cruzi*, but in *T. brucei* the knock-down of TXN1 reduced the growth BSF and procyclics and in *L. infantum* it was required for cell survival (Wilkinson et al., 2003; Comini et al., 2007; Romao et al., 2009). Given its central role in the antioxidant system and its absence in human cells, trypanothione reductase is considered an attractive drug target (Figure 2). Additionally, RNAi and knockout studies demonstrated that it is an essential gene in *T. brucei, L. donovani*, and *L. major*. Additionally, in *Leishmania* a single allele knockout of the gene resulted in reduced infectivity and capacity to survive within macrophages (Dumas et al., 1997; Tovar et al., 1998; Krieger et al., 2000). Although not directly involved in the antioxidant system, we also search data on the essentiality of the two enzymes required for the production T(SH)_2_ (Figure 2). In BSF of *T. brucei*, knockdown of TryS resulted in growth arrest and led to depletion of both T(SH)_2_ and its precursor glutathionylspermidine (Comini et al., 2004). In *L. infantum*, both promastigotes and amastigotes with deletion of GspS were viable. In contrast, elimination of both TryS alleles was only possible when parasites were previously complemented with an episomal copy of the gene (Sousa et al., 2014).

Drugs of current use for the treatment of diseases caused by *T. cruzi* and other trypanosomatids seems to have several modes of action (Field et al., 2017). There is some evidence that one of them is the induction of oxidative stress within the parasite, as pharmacological and genetic inhibition of its antioxidant defense mechanism have effects on the susceptibility to the drugs. For example, treatment of different *T. cruzi* stages with buthionine sulfoximine, an inhibitor of the synthesis of an indispensable precursor of glutathione, increase the antiparasitic effects of nifurtimox and benznidazole (Faundez et al., 2005). However, the molecular targets of these drugs have not been elucidated, as overexpression of some of antioxidant enzymes do not have an effect on drug sensitivity (Wilkinson et al., 2000). Similar to *T. cruzi*, some of the drugs currently used to treat leishmaniasis may also target the antioxidant system. The levels of expression of cTXNPx have been associated with drug resistance to antimonials and amphotericin B in several species of *Leishmania* (Suman et al., 2016; Das et al., 2017). Promastigotes of *L. donovani, L. tarentolae*, and *L. brazilensis*, but not *L. infantum*, overexpressing cTXNPx increased their resistance to antimonials (Iyer et al., 2008; Wyllie et al., 2008; Andrade and Murta, 2014; Das et al., 2017). On the other hand, there have also been efforts to discover and design drugs against enzymes of the antioxidant system, particularly for those involved in the trypanothione synthesis and recycling (Wyllie et al., 2009; Patterson et al., 2011; Spinks et al., 2012).

The components of antioxidant system of *T. cruzi* are attractive drug targets against the parasite, as many of them are essential and absent in mammals. Also, they apparently lack functional redundancy (Krauth-Siegel and Comini, 2008), making them highly selective. Additionally, drugs currently used to treat the diseases caused by trypanosomatids may act, at least in part, by interfering in the antioxidant system of the parasite. Identifying the precise molecular targets within this pathway may open the possibility of designing more potent and selective drugs.

### Synthesis of Surface Glycoconjugates

The GPI anchor is a glycolipid structure formed at the parasite endoplasmic reticulum (ER). This moiety has a conserved core of phosphatidylinositol (PI), linked to a trimannosyl-non-acetylated glucosamine. This sugar core is linked to an ethanolamine (Etn) phosphate that is attached to the C-terminus of the protein via an amide bond. The two fatty acids within the hydrophobic PI group anchor the protein to the cell membrane (Cheung et al., 2014). There can be a wide variety of substituents in the mannoside, inositol, or lipid moieties depending on the particular protein, organism, or developmental stage of an organism (Hong and Kinoshita, 2009). The GPI anchor is synthesized by a variety of enzymes, which sequentially transfer sugars and Etn to the PI. After synthesis, the GPI anchor is attached to the protein by GPI transamidase before being transported to the cell surface. The first two steps of the synthesis of GPI anchors that result in the production of the N-glucosaminyl-phosphatidylinositol (GlcNAc-PI) occur at the outside face of the ER membrane and then it is flipped to the inside. Similarly, dolichol-phosphate mannose (Dol-P-Man), the donor molecule for the mannoses within the GPI core, is synthesized in the outside face of the ER membrane, and then flipped inside. The rest of the synthesis of the GPI core occurs inside the ER.

**Figure 3:**
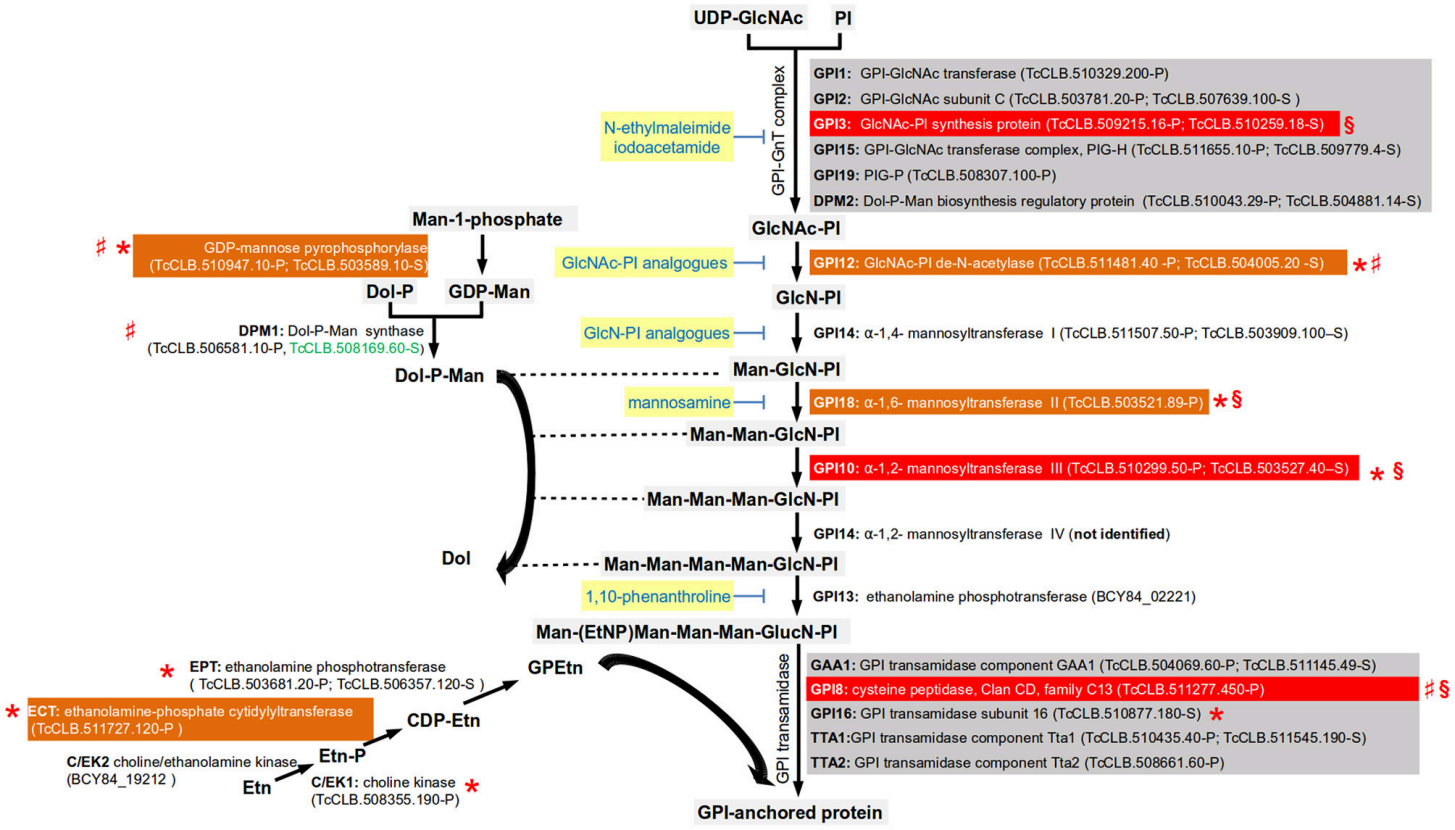
Simplified pathway for the synthesis of the GPI-core (based on Cardoso et al., 2013). Gene names are annotated with bold letters, followed by their current annotation in the TriTrypDB. In brackets are the *T. cruzi* locus identifiers of complete genes, followed by the haplotype to which they belong (P = Non-Esmeraldo; S = Esmeraldo). Sequences that have been identified as incomplete or pseudogenes are not included. Because the *T. cruzi* CL Brener sequences for GPI13 are incomplete for both haplotypes (TcCLB.503979.10-P and TcCLB.507667.11-S), the ID for the complete GPI13 gene for the *T. cruzi* Dm28c is used. Similarly, for the choline/ethanolamine kinase 2, for which both CL-Brener alleles encode for pseudogenes (TcCLB.487739.20-P; TcCLB.508805.30-S/TcCLB.511181.160-S) and therefore the full sequence from the *T. cruzi* Dm28c strain is used. Sequences classified as highly druggable (druggability index >0.7) according to the Target-Pathogen database (http://target.sbg.qb.fcen.uba.ar/patho/) are shown with green letters. Known drug classes that block enzymes within this pathway are identified by yellow boxes with blue letters. With a red box are highlighted the enzymes known to be essential in *T. cruzi*, and in orange boxes are highlighted enzymes that are essential in other trypanosomes. Enzymes that have been genetically manipulated to evaluate their potential role as therapeutic targets in *T. cruzi* (§), *T. brucei* (*), or *Leishmania* (♯) have been annotated with a symbol.

All the enzymes involved in the *T. cruzi* GPI biosynthetic pathway have been identified, with the exception of the enzyme required for the addition a fourth mannose into the GPI core (Cardoso et al., 2013; Figure 3). Several differences between the mammalian and trypanosmatid pathways were identified. The mammalian GPI-N-acetylglucosaminyltransferase complex (GPI-GnT) has an element known as PIG-Y that is not present in trypanosomatids (Murakami et al., 2005). There are also differences in the composition of GPI transamidase, the enzymatic complex in charge of adding and processing the protein to the GPI core. The parasitic and the mammalian enzymes have 5 elements, but only three are shared in both complexes (GAA1, GPI8, and PIG-T) and the two others are specific of the mammalian host (PIG-S and PIG-U) or of the parasite (trypanosomatid transamidase 1 and 2) (Nagamune et al., 2003). Also, mammalian cells require the acylation of the inositol ring prior to the addition of the first mannose to the non-N-acetylated glucosaminyl (GlcN)-PI by the enzyme PIG-W. This acylation group is removed after the addition of the sugar residue to the core and the attachment of the GPI anchor to the protein (Cardoso et al., 2013). No orthologs of PIG-W were identified in either *T. cruzi* or *T. brucei*. However, orthologs involved in inositol deacylation are present in the *T. cruzi* genome (Figure 3), as well as in *T. brucei* and *Leishmania*. Mammalian GPI has extra ethanolamine phosphate groups (Etn-P) in other residues, reactions that are catalyzed by different enzymes. The enzymatic pathway that synthesize the donor for ethanolamine group have been identified in *T. brucei* and their orthologs can be identified (Gibellini et al., 2008). Reverse genetics have been used to assess the potential of this biosynthetic pathway as a therapeutic target identifying some differences between species that deserve further study. In *T. brucei*, RNAi studies have demonstrated that GPI8 (Lillico et al., 2003), GPI10 (Nagamune et al., 2000), and GPI12 (Chang et al., 2002) are essential for the bloodstream form (BSF) of the parasite. In contrast, in procyclic cells these enzymes are not essential for growth *in vitro* and therefore knockouts of both alleles have been achieved. GPI8, GPI10, and GP12 procyclic knockouts have an abnormal surface glycocalix that do not allow these cells to colonize the tsetse midgut (Nagamune et al., 2000; Güther et al., 2006). The knockout of GPI16 (the ortholog of the human PIG-T) in procyclic cells had normal morphology but reduced growth rate (Hong et al., 2006). In *Leishmania major*, the GPI12 knockout has been obtained in the promastigote stage. Although viable, the knockout had decreased ability to infect and multiply into murine macrophages (Almani et al., 2016). In *L. mexicana*, GPI8 null mutants were obtained that grew normally in liquid culture, and were able to successfully infect macrophages *in vitro* and mice (Hilley et al., 2000). In *T. cruzi* epimastigotes, it was not possible to obtain null mutants for GPI3, GPI8, and GPI10 by homologous recombination suggesting that these enzymes are essential. Interestingly, it was not even possible to obtain a single allele knockout of GPI3 and GPI10 suggesting the need for both functional alleles of the genes (Cardoso et al., 2013).

The pathways involved in the synthesis of mannose and Etn donors for GPI synthesis are also required for parasite survival (Figure 3). For the synthesis of Dol-P-Man, the GDP-mannose pyrophosphorylase was found to be essential for the BSFs of *T. brucei* (Denton et al., 2010). In *L. mexicana* procyclic forms, knockouts of either GDP-mannose pyrophosphorylase, or Dol-P-Man synthase were viable but demonstrated decreased virulence (Garami and Ilg, 2001; Garami et al., 2001; Stewart et al., 2005). With respect to glycerophosphoethanolamine (GPEtn) synthesis, the locus annotated as encoding for the enzyme involved in the first step of the pathway (the choline/ethanolamine kinase, C/EK) contains only pseudogenes in the *T. cruzi* CL-Brener genome (Figure 3). Interestingly, analysis of synteny of the orthologs in other *T. cruzi* strains reveals the presence of full length open reading frames. The absence of a complete gene in one strain could suggest that the enzyme is not essential for the parasite. However, in *T. brucei* two choline/ethanolamine genes are present, being initially named C/EK1 and C/EK2 (Gibellini et al., 2008). *In vitro* characterization of their catalytic activities demonstrated that C/EK1 metabolize Etn but not choline, and therefore they rename it TbEK1. In contrast, TbC/EK2 metabolized both substrates. In the *T. cruzi* CL-Brener genome there is only a functional ortholog for TbEK1, with the locus encoding for the putative TbC/EK2 being a pseudogene. Thus, it is important to characterize the catalytic abilities of the ortholog to TbEK1. In *T. brucei* the three enzymes involved in the synthesis of GPEtn have been evaluated in the procyclic form by reverse genetics (Gibellini et al., 2008, 2009; Signorell et al., 2009). RNAi of the three enzymes resulted in viable organisms. However, in the case ethanolamine-phosphate cytidylyltransferase (ECT), depletion of the enzyme was associated with abnormal mitochondrial morphology, and with the accumulation of multinucleated cells (Signorell et al., 2009). A TbECT conditional null mutant of the BSF was obtained by homologous recombination associated with the expression of an ectopic copy of the gene demonstrated that this gene is essential (Gibellini et al., 2009).

Several inhibitors of the GPI synthesis have been shown to kill trypanosomatids. Analogs of the biosynthetic pathway frequently inhibit both parasite and mammalian enzymes. However, different type of substitutions have been evaluated to obtain greater specificity for the trypanosomatid enzymes. Inhibitors that affect GPI synthesis in trypanosomatids without affecting the mammalian enzymes need to be found.

#### Glycosylation Steps Evaluated as Potential Drug Targets

The specific type of glycoconjugates present in *Trypanosoma cruzi, T. brucei*, and *Leishmania major*, are fundamentally different, reflecting their disparate life cycles, modes of infection, and disease pathologies (Turnock and Ferguson, 2007). The addition of sugar moieties to their targets occurs in the lumen of the ER and Golgi apparatus through the action of glycosyltransferases using nucleotide sugars as substrates. Translocation of the sugar nucleotide from the cytosol into the Golgi lumen is mediated by a transporter specific for each nucleotide sugar. Ten different sugar nucleotides have been identified in trypanosomatids, but the repertoire present in each parasite is species-specific (Turnock and Ferguson, 2007). Five nucleotide sugars are common to the three species [GDP-α-D-mannose, UDP-α-D-N-acetylglucosamine, UDP-α-D-glucose, UDP-α-galactopyranose (UDP-Gal*p*), and GDP-β-L-fucose], three are identified only in *T. cruzi* (UDP-β-L-rhamnopyranose, UDP-α-D-xylose, and UDP-α-D-glucuronic acid), GDP-α-D-arabinopyranose is found only in *L. major*, and UDP-α-D-galactofuranose (UDP-Gal*f*) is present in *T. cruzi* and in *L. major* (Turnock and Ferguson, 2007). Mammalian cells do not synthetize UDP-α-D-galactofuranose and therefore its biosynthetic pathway is an attractive target for new drugs.

**Figure 4:**
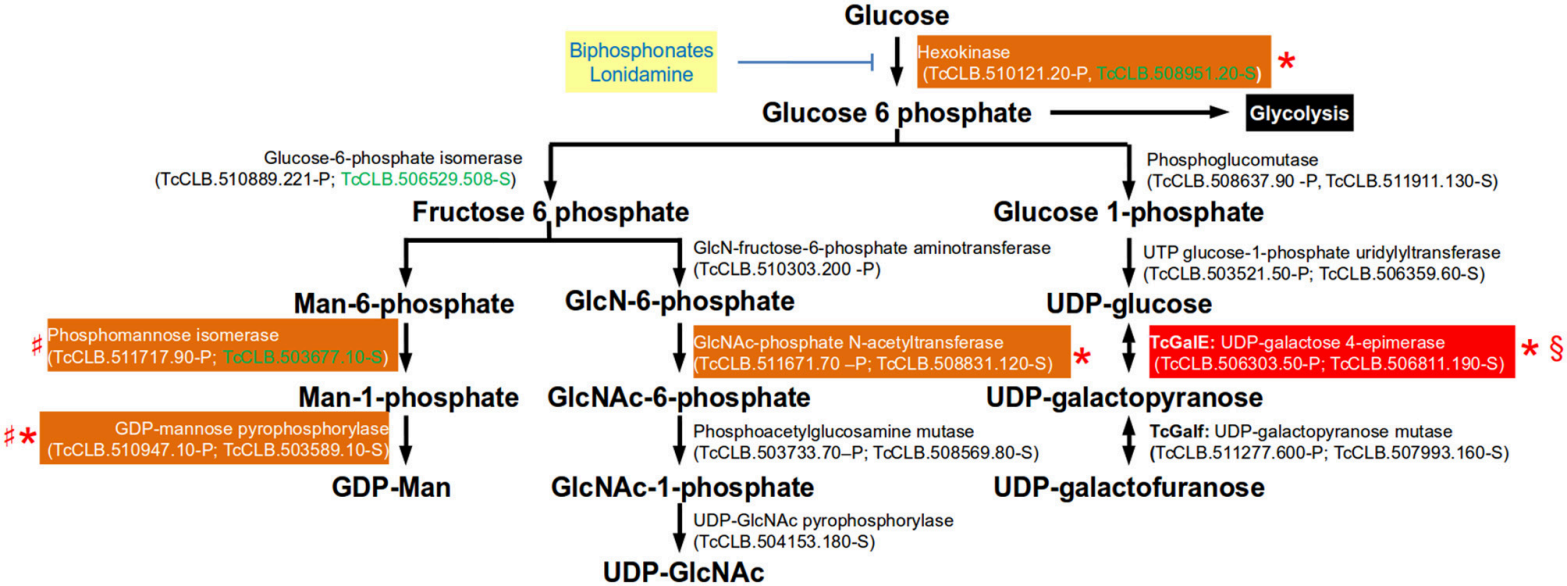
Glycosylation pathways where essential genes have been identified in trypanosomatids. Genes are identified by their current annotation in the TriTrypDB. In brackets are the *T. cruzi* locus identifiers of complete genes, followed by the haplotype to which they belong (P = Non-Esmeraldo; S = Esmeraldo). Sequences that have been identified as incomplete or pseudogenes are not included. Sequences classified as highly druggable (druggability index >0.7) according to the Target-Pathogen database (http://target.sbg.qb.fcen.uba.ar/patho/) are shown with green letters. Drugs that block hexokinase are highlighted in a yellow box with blue letters. With a red box are highlighted the enzymes known to be essential in *T. cruzi*, and in orange boxes are highlighted enzymes that are essential in other trypanosomes. Enzymes that have been genetically manipulated to evaluate their potential role as therapeutic targets in *T. cruzi* (§), *T. brucei* (*) or *Leishmania* (♯) have been annotated with a symbol.

To act as glycosyl donors, the monosaccharides must be conjugated with a nucleotide. In trypanosomatids the biosynthesis of all nucleotide sugars initiate with glucose-6-phosphate, with the exception of GDP-arabinose (present only in *Leishmania* spp.) which is thought to be synthesized from glucose by an unknown mechanism (Turnock and Ferguson, 2007). The synthesis of glucose-6-phosphate from glucose is therefore not only important in glycolysis, but also for the synthesis of sugar nucleotides in *T. cruzi* (Figure 4). Hexokinase catalyzes the conversion of the glucose that has entered the cell into glucose-6-phosphate. The regulation of *T. cruzi* hexokinase differs from the mammalian enzyme in that is not inhibited by glucose-6-phohosphate, fructose-1,6-diphosphate, phosphoenol pyruvate, malate, or citrate. Instead, the parasite enzyme, but not the human, is inhibited by non-hydrolyzable analogs of inorganic pyrophosphate (biphosphonates). Biphosphonate inhibitors of the *T. cruzi* enzyme that are not toxic to human cell lines have been identified (Hudock et al., 2006). It was proposed that the lack of toxicity depends of the specificity of the analogs which are highly specific of the hexokinase and poor inhibitors of farnesyl diphosphate synthase (enzyme that is involved in the Ergosterol synthesis pathway, see Figure 1). Lonidamine is another inhibitor that reduces viability of *T. cruzi, T. brucei*, and *Leishmania* (Chambers et al., 2008).

In *T. cruzi*, the major surface glycoproteins are mucin-like proteins which are heavily O-glycosylated. A distinctive property of this type of glycosylation in this organism is that the sugar attached to the hydroxyl group of serine or threonine residues is GlcNAc, whereas both *T. brucei* and *Leishmania* attach GalNAc. The *T. cruzi* and *T. brucei* hexose transporters do not transport D-galactose and therefore their only source of galactose is through the epimerisation of UDP-Glc to UDP-Gal by the UDP-glucose 4'epimerase (GalE) (Roper and Ferguson, 2003; Figure 4). Unlike the mammalian enzyme, the parasitic enzyme is unable to interconvert UDP-GlcNAc and UDP-GalNAc. Therefore, GalE is essential for the synthesis of UDP-Gal*p* and UDP-Gal*f* abundant residues in the *T. cruzi* mucins. The conversion of UDP-Gal*p* to Gal*f* depends on the activity of UDP-Gal*p* mutase, and this enzyme present only in *T. cruzi* and *Leishmania* but not in mammals, which makes it an interesting therapeutic target (Oppenheimer et al., 2011). The attachment of mucins to the plasma membrane is through a GPI-anchor and therefore synthesis of UDP-Man is essential.

Terminal β-Gal*p* residues of *T. cruzi* mucins are sialylated by a GPI-anchored trans-sialidase that catalyzes the transfer of the host's sialic acid to the parasite's proteins. Sialylation of mucins is important for the survival for *T. cruzi*. In epimastigotes it has been implicated adhesion of the parasites to the epithelial cells in the rectal ampoule of the insect. In trypomastigotes, the terminal sialic acids mask parasite antigenic determinants, thus protecting the parasite from host attack by anti-galactosyl antibodies and by complement (Giorgi and de Lederkremer, 2011). Inhibitors of the trans-sialidase are being developed as potential therapeutic agents. Reverse genetics studies have demonstrated the importance of these pathways *in vivo* (Figure 4). In *T. brucei* there are two hexokinase genes in tandem encoding for proteins with hexokinase that are 98% identical. Experiments with RNAi and ectopic expression of the enzyme demonstrated that hexokinase activity is essential for *T. brucei* procyclic and BSFs (Albert et al., 2005; Chambers et al., 2008). Within the biosynthetic pathway for UDP-Gal*p*, tetracycline-inducible conditional *GalE* null mutants of *T. brucei* procyclic and BSFs were found to be essential (Roper et al., 2002, 2005). This also seems to be the case for *T. cruzi* epimastigotes as it was not possible to obtain the double allele knockout (MacRae et al., 2006). Unlike *T. cruzi* and *T. brucei, Leishmania* can obtain galactose from extracellular sources, and therefore GalE is not essential in these parasites (Oppenheimer et al., 2011). In *L. major*, double knockout of UDP-galactopyranose mutase (*Galf*) has been obtained. The null mutants were viable but had decreased virulence in mice (Kleczka et al., 2007). In *T. cruzi*, the targeting of two alleles encoding a conserved hypothetical protein (TcCLB.511301.50-P;TcCLB.511353.30-S) that was considered a possible triose-phosphate or UDP-galactofuranose transporter (Galf-transpoter) was found to be essential (Soares Medeiros et al., 2017). However, although the encoded protein could correspond to a sugar nucleotide transporter, there is no evidence that the transported sugar-nucleotide is indeed Galf. As this gene proved to be essential it would be important to do a full characterization of the encoded protein. The role in viability of all enzymes involved in the synthesis of UDP-Man in *Leishmania* have been studied. Promastigote mutants null for phosphomannose isomerase, phosphomannose mutase, and GDP-Man pyrophosphorylase are all viable, and with the exception of the phosphomannose isomerase, the mutants lost their ability to infect macrophages and mice (Turnock and Ferguson, 2007). As previously mentioned, the GDP-mannose pyrophosphorylase is essential in *T. brucei* (Denton et al., 2010). Reverse genetics in the pathway of UDP-GlcNAc synthesis has only been determined for the glucosamine 6-phosphate n-acetyltransferase of *T. brucei*. In BSFs of *T. brucei* the conditional null mutant was unable to sustain growth under non-permissive conditions, demonstrating that there are no metabolic, or nutritional routes to UDP-GlcNAc other than via GlcNAc-6-phosphate (Mariño et al., 2011).

## Conclusions and Perspectives

New treatments for Chagas' disease are required. More information is needed not only of the molecular mechanisms involved in the establishment of infection, but also about those involved in parasite survival and persistence. The screening of chemical libraries will undoubtedly identify new potential targets, but they will also need to be individually curated and validated. Unfortunately, it is still difficult to prove the essentiality of a gene in *T. cruzi*, although the CRISPR-Cas9 technology appears to be more efficient for gene disruption than the knockouts by homologous recombination. Improved strategies for the regulated expression of ectopic genes needs to be developed. In the meantime, it is always useful to assess the information available for other trypanosomatids. Public databases such as TriTrypDB or TDR Targets present a great opportunity for data mining and contain a wealth of information that can be easily searched and analyzed. However, the genome sequence of the parasite genome still contain gaps and some errors, so integrity of the selected genes should always be assessed. Although molecular modeling of *T. cruzi* proteins can be done from structural information from orthologs of other species, to develop highly selective inhibitors to target proteins it is necessary to obtain crystal structures of the specific proteins. There is no doubt that identifying and developing new treatments requires a large team of specialists in multiple areas such as chemistry, biology, engineering, informatics, and medicine. Ideally, several teams should be assembled and organized so that every aspect of drug development is met and, hopefully, each team should concentrate in different potential targets.

## Author Contributions

JO-M and AC contributed equally in this work.

### Conflict of Interest Statement

The authors declare that the research was conducted in the absence of any commercial or financial relationships that could be construed as a potential conflict of interest.

## Supplementary Material

Supplementary Figure 1 Expression profile of selected genes obtained by RNA-seq during experimental infection of fibroblasts with *T. cruzi* (Li et al., 2016). Data was downloaded from the expression section of the TritrypDB. Data is expressed as transcript levels of fragments per kilobase of exon model per million mapped reads (FPKM). Red boxes represent unique transcript, gray boxes represent reads of sequences shared with other genes.
