# Supplementary figures and images for: Discovery and Genetic Validation of Chemotherapeutic Targets for Chagas' Disease
Source: Front Cell Infect Microbiol. 2019 Jan 7;8:439. doi: 10.3389/fcimb.2018.00439 (PMC6330712; doi:10.3389/fcimb.2018.00439)

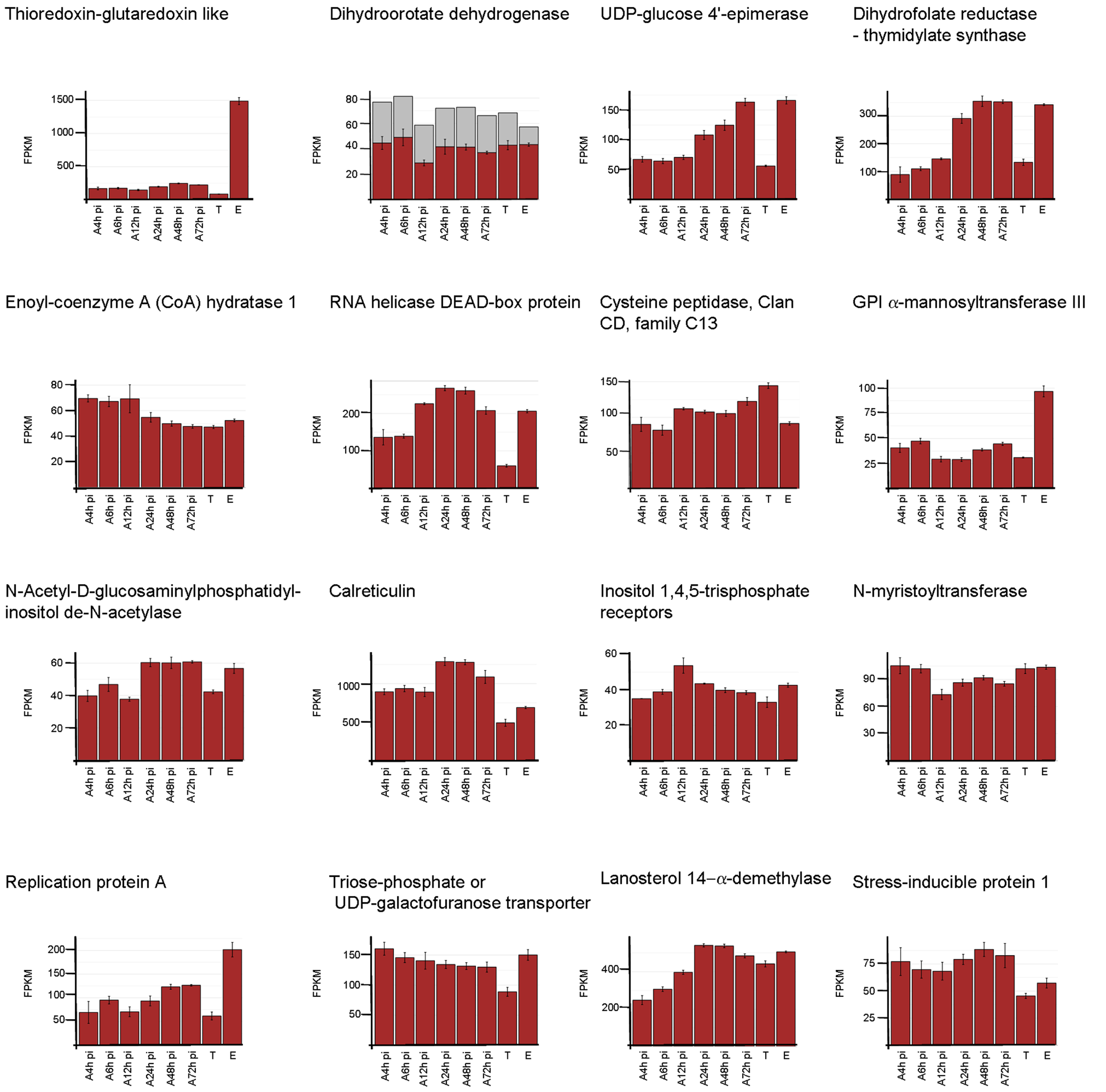

Supplement: Supplementary Figure 1 — Expression profile of selected genes obtained by RNA-seq during experimental infection of fibroblasts with T. cruzi (Li et al., 2016). Data was downloaded from the expression section of the TritrypDB. Data is expressed as transcript levels of fragments per kilobase of exon model per million mapped reads (FPKM). Red boxes represent unique transcript, gray boxes represent reads of sequences shared with other genes. [file Image_1.TIF]
